# Chemoresistance-Related Stem Cell Signaling in Osteosarcoma and Its Plausible Contribution to Poor Therapeutic Response: A Discussion That Still Matters

**DOI:** 10.3390/ijms231911416

**Published:** 2022-09-27

**Authors:** Sara R. Martins-Neves, Gabriela Sampaio-Ribeiro, Célia M. F. Gomes

**Affiliations:** 1 iCBR-Coimbra Institute for Clinical and Biomedical Research, Faculty of Medicine, University of Coimbra, 3000-548 Coimbra, Portugal; 2 Institute of Pharmacology and Experimental Therapeutics, Faculty of Medicine, University of Coimbra, 3000-548 Coimbra, Portugal; 3 CIBB-Center for Innovative Biomedicine and Biotechnology, University of Coimbra, 3000-548 Coimbra, Portugal; 4 CACC-Clinical Academic Center of Coimbra, 3000-075 Coimbra, Portugal

**Keywords:** osteosarcoma, cancer stem cell, chemoresistance, drug efflux, apoptosis, cell cycle, hypoxia, inflammation, metabolism

## Abstract

Osteosarcoma is amongst the most prevalent bone sarcomas and majorly afflicts children and adolescents. Therapeutic regimens based on the triad of doxorubicin, cisplatin and methotrexate have been used as the state-of-the-art approach to clinical treatment and management, with no significant improvements in the general outcomes since their inception in the early 1970s. This fact raises the following problematic questions: Why do some patients still relapse despite an initial good response to therapy? Why do nearly 30% of patients not respond to neoadjuvant therapies? Does residual persistent disease contribute to relapses and possible metastatic dissemination? Accumulating evidence suggests that chemoresistant cancer stem cells may be the major culprits contributing to those challenging clinical outcomes. Herein, we revisit the maneuvers that cancer stem cells devise for eluding cell killing by the classic cytotoxic therapies used in osteosarcoma, highlighting studies that demonstrate the complex crosstalk of signaling pathways that cancer stem cells can recruit to become chemoresistant.

## 1. Introduction

Osteosarcoma is very aggressive bone tumor with a dismal prognosis for poor responders to therapy and for metastasis-presenting patients [1]. Considering the histological observations of the existence of undifferentiated cellular subsets alongside with cells resembling more differentiated phenotypes, some studies propose a stem cell origin for osteosarcoma, including the possibility of tumor-promoting genetic hints that provide a hurdle to mesenchymal stem cells (MSCs) [2]. In fact, regardless of its origin, a plethora of evidence demonstrates that cancer stem cells (CSCs) exist within osteosarcomas and have a role as promising therapeutic targets [3].

Despite the difficulties in the identification of a consistent molecular phenotype for osteosarcoma CSCs, knowing the mechanisms by which stemness networks persist among tumor cells is particularly important for the recognition of new therapeutic targets [4]. Unpuzzling the signaling pathway players that determine chemoresistance and how they are molecularly intertwined with stemness signaling offers the possibility of effectively enhancing chemosensitivity for osteosarcoma. Therefore, in this review, we focus on the key mechanisms involved in resistance to chemotherapy in osteosarcoma CSCs, namely, detoxification systems (drug efflux transport and ALDH), survival-related pathways (ERK, AKT), adaptive metabolic routes, altered cell cycle and DNA repair, enhanced apoptosis and modulation of the tumor microenvironment (hypoxia, inflammation), by giving appropriate examples. We also discuss a few points regarding some of the immunotherapeutic options explored for osteosarcoma treatment, with a tentative focus on the few studies that specifically regard CSCs.

## 2. Epidemiology of Osteosarcoma and Rationale to Maintain the Discussion about Its Resistance to Chemotherapy

**Figure 1:**
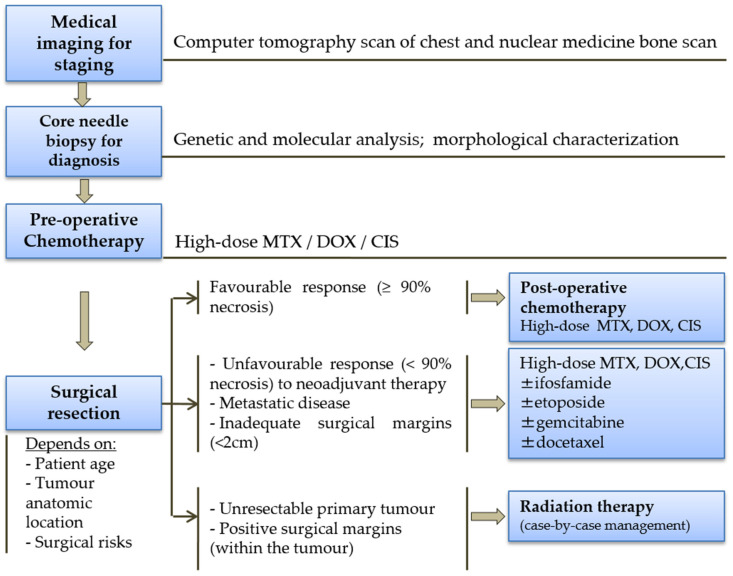
Osteosarcoma therapeutic management. Doxorubicin (DOX), cisplatin (CIS) and methotrexate (MTX) are the first-line and the main chemotherapeutic drugs used in the treatment of osteosarcoma (diagram compiled from [6,11,12].

Osteosarcoma is the commonest type of bone cancer and a highly aggressive osteoid-depositing tumor affecting mainly children and adolescents [5]. Nearly 20% of patients have metastatic disease at presentation, mostly located in the lungs [6]. The treatment for osteosarcoma follows a multidisciplinary approach (Figure 1), with the essential standard therapy including (a) pre-operative or neoadjuvant chemotherapy, consisting of high-dose methotrexate (MTX), doxorubicin (DOX) and cisplatin (CIS); (b) local surgical resection; and (c) post-operative or adjuvant chemotherapy, administered when the degree of tumor necrosis after pre-operative chemotherapy is superior to 90% [7]. This chemotherapeutic regimen has improved the cure rate and long-term disease-free survival percentage for osteosarcoma patients, with localized lesions ranging from 5% to 20% in the pre-chemotherapy era to the 65% to 75% range observed nowadays [7,8,9]. Unfortunately, survival for patients with metastasis at initial diagnosis is still only 17–34% [10].

Pre-operative chemotherapy enables the early treatment of micrometastatic disease and facilitates the surgical resection by shrinking the tumor mass and decreasing vascularity. Limb-salvage surgery should occur after a defined time interval, with no advantage being observed with immediate surgery [13], aiming to preserve a functioning limb without increasing the risk of post-operative complications to the patient. Post-operative chemotherapy after surgical resection is normally performed in order to minimize the likelihood of local recurrence. It is clinically well-established that complete surgical resection of both the primary tumor and metastatic nodules is essential for survival [6,10].

Despite a good response to pre-operative therapy, local recurrence and metastatic disease occurs in 25–50% of surgically-treated patients without evidence of metastasis at diagnosis. This is most probably attributable to poor response to standard therapy and constitutes the major clinical problem: preventing the curing of high-grade osteosarcoma patients. Patients who develop metastatic disease after surgical resection have limited options; however, they frequently receive additional cytotoxic drugs to the standard drug regimen, such as zoledronate [14], gemcitabine and docetaxel [15], ifosfamide [16], regorafenib [17] or lenvatinib [18]. Nevertheless, in general, in these patients, neither the intensification of dose regimens [19,20,21,22] nor the addition of new drugs [23,24] has significantly modified their clinical outcomes.

**Figure 2:**
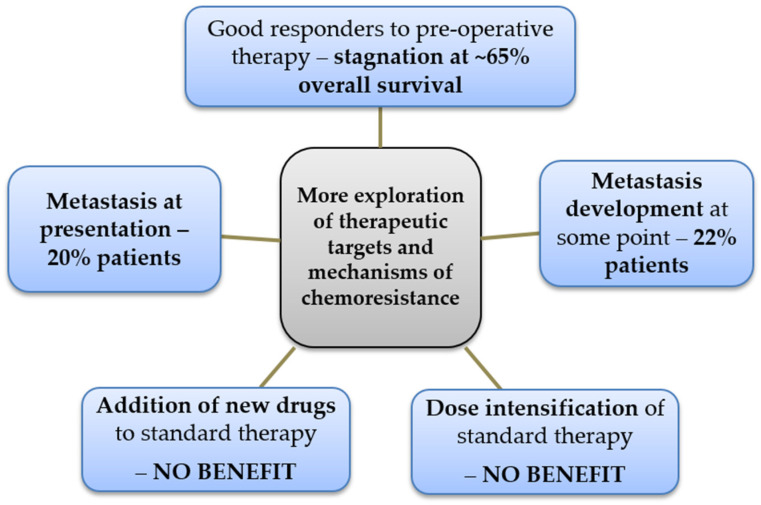
Rationale for the continuous discussion of the mechanisms of chemoresistance in osteosarcoma, derived from the plateaued outcomes and lack of benefits from the therapeutic interventions attempted in the modern clinical era (new drugs’ addition and dose intensification).

Notwithstanding the exhaustive and incredible progress in the clinical research of new therapeutic targets and compounds in recent years, the attempts to increase the proportion of tumor necrosis by means of dosage intensification of pre-operative chemotherapy and the addition of new compounds to the classical triplet drug regimen (MTX, DOX and CIS), the cure rate and long-term disease-free survival percentage of osteosarcoma patients with localized disease has stagnated since the 1970s, in the 60% to 70% range, as previously indicated [25,26]. The cases of disease recurrence and the development of drug resistance in osteosarcoma provide a rationale for the exploration of both old and new insights that might contribute to a better identification of the precise mechanisms of resistance operating in this tumor and the pathways responsible for recurrence after a favorable response to therapy (Figure 2).

## 3. Cancer Stem Cells Contribute to Intra-Tumor Cellular Heterogeneity

Tumor cell heterogeneity is a well-recognized characteristic of osteosarcoma, constituting one of the main causes of treatment failure and may have at least two underlying causative tumor development models—the stochastic clonal evolution and the hierarchical cancer stem cell (CSC). The existence of different osteosarcoma clones is linked to dynamic genetic and epigenetic events, which subsequently determines differential patterns of cellular tumorigenicity [27,28]. However, analysis of histopathological specimens reveals that, in some cases, the tumors are organized in a hierarchical manner, with a leading CSC being the generator of phenotypic and functional heterogeneity. In fact, cells with stemness-related features have been found in several tumor types, including bone sarcomas [29,30], and are associated with treatment resistance, tumor relapses and recurrences, and disseminated metastatic disease [31]. In recent years, several studies suggest that the principles of both the clonal and the CSC models can be conciliated to better describe tumor heterogeneity, since virtually every cell within the tumor may convert from a non-tumorigenic to a tumorigenic state and is able to switch phenotypically into a stem-like cell. These transitions are sustained by the appropriate oncogenic insults or other microenvironmental stimuli, demonstrating the CSC’s intrinsic cell plasticity [32,33,34,35].

### General Overview about the Identification of CSCs in Osteosarcoma

The identification of osteosarcoma CSCs has gained increasing attention over the last three decades. Owing to CSCs’ rarity and the absence of established specific markers for osteosarcoma, the characterization of CSCs has been done mostly based on functional criteria (Table 1), namely, with sphere-formation, Aldefluor™, side-population and surface marker flow cytometry-based assays. These methods do not mandatorily recognize a unique, exclusive set of CSCs, but do uncover the heterogeneous nature and phenotypic plasticity of osteosarcoma CSC sub-populations, with no methodology being better or more adequate than the other to identify those CSC subsets. In fact, based on our own previous experience with these techniques, when possible, combining, e.g., two of these functional assays may contribute to refine more bona-fide CSC subsets within the cell samples analyzed.

Cancer stem cell markers are used as permanent labels of stemness during self-renewal. Virtually every protein can serve as CSC biomarkers; however, not all are able to specifically identify a CSC population, as they might differ according to the source of CSCs and might change as the tumor evolves. Moreover, stem cells of different tissues are not all identical and the dissimilarities concerning, e.g., location, self-renewal and differentiation capabilities are often reflected by specific combinations of phenotypic markers.

Different combinations of markers constitute the basis for distinguishing a certain stem cell type from another one. Moreover, CSCs’ markers expression might be influenced by intrinsic tumor microenvironmental stimuli or modulated by therapeutics, as we previously discussed [74]. There is consensus that CSCs express many of the markers commonly used to identify normal stem cells (either embryonic or adult somatic stem cells) [75]. In general, these cell surface markers are very advantageous to identify and isolate CSC populations using the appropriate cell-sorting technologies and protocols.

Some markers are inclusive—that is, expressed by diverse types of CSCs—or exclusive, with the potential to be exploited for therapeutic targeting or as biomarkers. The first specific CSC markers were identified in hematological tumors, CD34+/CD38- in acute myeloid leukemia [76], while breast cancer was the first solid tumor for which a CD44+/CD133+/ALDH1+ phenotype was pointed as specific for breast CSCs [77,78].

Comprehensible descriptions of osteosarcoma CSC markers have been revised previously [79,80]. Notably, none of the herein- and therein-mentioned markers are unique to osteosarcoma CSCs. Indeed, CD surface markers presented in Table 1 either require a broader validation in osteosarcoma tissue cohorts or they have been detected also in other CSC types; for example, CD44/CD271 in melanoma [81] and CD117 in ovarian cancer [82]. Moreover, the identification of CSC surface markers has been more elusive in mesenchymal tumors than in tumors originating from other tissue types, in part due to the lack of agreement on the markers that unequivocally identify mesenchymal progenitor cells [83], the plausible osteosarcoma cells of origin [84]. Nevertheless, research towards specific molecular markers for osteosarcoma CSCs that may be therapeutically targeted is ongoing and justifies further investigation.

## 4. Mechanisms of CSC Resistance to Conventional Therapies

**Figure 3:**
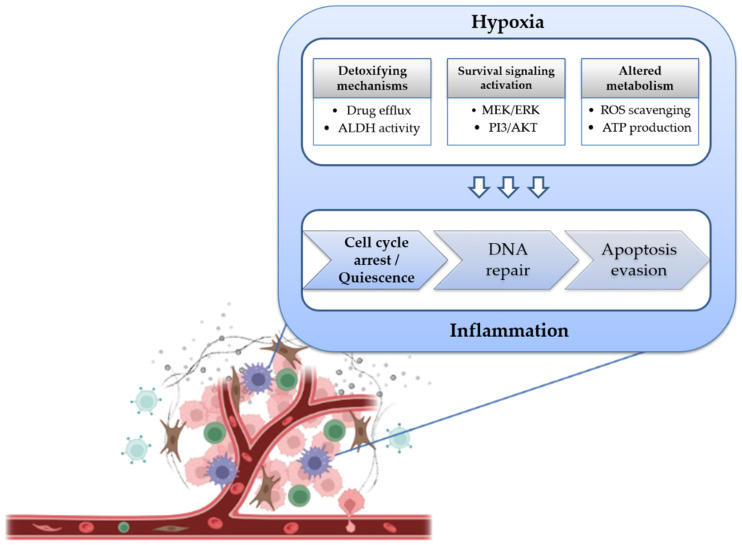
Overview of the mechanisms of chemoresistance in osteosarcoma CSCs highlighted in this review. Supportive tumor microenvironmental conditions, modulated by hypoxia and inflammation, cooperate with detoxifying mechanisms, survival pathways activation and altered metabolism to induce a quiescent state, allowing DNA repair activation and culminating in evasion from apoptotic cell death.

Several studies using cancer cell lines and pathological tissue specimens indicate that a complex network of mechanisms play a determinant role in cancer cell resistance to chemotherapeutics. The knowledge of the different chemoresistance-related pathways in osteosarcoma CSCs is then vital for the development of novel molecular targets that may enhance their chemosensitivity. Some of the mechanisms explored in this review seem exacerbated in osteosarcoma CSCs, which can undertake a quiescent state and activate signaling cascades, such as drug efflux transport or aldehyde dehydrogenase activity, and that may accompany apoptosis evasion, enhanced survival and DNA repair activation. Moreover, hypoxic and pro-inflammatory microenvironments surrounding CSCs also constitute facilitators of resistance to conventional therapies that target rapidly proliferating cells and induce DNA damage (Figure 3).

### 4.1. Chemoresistance Due to Detoxifying Mechanisms—Drug Efflux Transporters and Aldehyde Dehydrogenase Activity

#### 4.1.1. Drug Efflux Transporters

Cancer cells can become resistant to multiple cytotoxic drugs through increased efflux of the drug from the cell, via the so-called ABC transporters. Overexpression of these molecular membrane pumps contributes to multidrug resistance, as they export a wide variety of drugs, including doxorubicin, cisplatin and methotrexate, which are clinically used in osteosarcoma. Multidrug resistance (MDR) may cause relapses to therapy, which together with metastatic dissemination is still a major contributor to death by cancer [85]. High expression of ABC transporters has been detected in several CSC types and correlated with resistance to diverse chemotherapeutics [86]. The study of the impact of ABC transporters in bone tumors has regained attention in recent years, since they are expressed by both normal tumor cells and CSCs, with ABCB1 and ABCG2 being of special interest. In fact, particularly ABCB1 expression has been positively correlated to the existence of metachronous lung metastases and a propensity to metastatic formation in non-responsive patients, as previously reviewed [87].

The expression and function of several ABC transporters seems to be exacerbated in osteosarcoma CSCs and linked to chemoresistance. ABGC2-high CSCs selected with 3-aminobenzamide had increased drug efflux ability [36]. We showed that the ABC inhibitor verapamil enhanced the cellular uptake of doxorubicin by P-glycoprotein- and BCRP-overexpressing CSCs [40]. This was encompassed by Bak upregulation and Bcl-2 suppression favoring CSC apoptosis [88]. We also found a positive correlation between *ABCG2* expression and a side-population cell subset in nine cell lines [41]. Moreover, doxorubicin induced ABCG2 and ABCB1 expression paralleled by the activation of pluripotency markers and Wnt/β-catenin in bulk cells [50], while the inhibition of tankyrase with IWR-1 in CSC-enriched spheres led to downregulation of BCRP and P-glycoprotein, accompanied by a sensitization to doxorubicin-induced apoptosis [89]. Other studies showed that cisplatin-resistant CD133+ MG-63 cells express high levels of P-glycoprotein [90], and the same was observed in doxorubicin-resistant U2OS and MG-63 spheres [91]. Moreover, elevated ABCG1 and P-glycoprotein expression was found in doxorubicin-selected CSCs and in progeny derived from a single HOS cell, mediating resistance to doxorubicin and other drugs [92]. In 3D cultures of ABCB1-high/ABCA1-low chemo-immune-resistant cells, expression of the doxorubicin effluxer ABCB1 was upregulated by the Ras/ERK1/2/HIF-1α signaling axis, which suggests the existence of pathway crosstalk to reinforce an already chemoresistant phenotype in osteosarcoma cells [93]. ABCG2 transcriptional activity was also suppressed by the transcriptional regulator SMAR1, which increased the ABCG2 deacetylation level via HDAC2 and also decreased the ALDH activity [94]. miRNA-221 also seems to increase P-glycoprotein expression via the Stat3 pathway and promoting doxorubicin-resistance [95].

The modulation of ABC transporters activity to circumvent chemoresistance in osteosarcoma has been difficult to translate into successful clinical achievements, with controversial data being reported regarding ABCs as prognostic factors or significantly related to outcomes, as reviewed before [96]. However, considering that the first-line chemotherapeutics that are still indispensably used in osteosarcoma (doxorubicin and cisplatin) are substrates of several drug efflux pumps, revisiting their role as major contributors to MDR [97], exploring their pharmacogenetic and pharmacogenomic association with osteosarcoma survival and response to therapy [98] and admitting that ABCs may also be detrimental to osteosarcoma CSC survival, may altogether refine and renovate their clinical applications.

#### 4.1.2. Aldehyde Dehydrogenase Activity

A plethora of studies have investigated the role of ALDH expression and activity in diverse tumor types and revealed that expression of ALDHs is involved in disease progression and metastatic dissemination [99], including osteosarcoma. ALDHs are actively involved in the chemoresistance of CSCs and their expression generally correlates with a poor prognosis [100]. ALDH enzymes mediate the conversion of xenobiotic and intracellular aldehydes, such as drugs, ethanol and vitamins, into their less harmful corresponding carboxylic acids, thereby acting as important mediators of defense against cytotoxic compounds that can induce DNA damage, inactivation of enzymes and even cell death. ALDHs are responsible for the metabolic regulation of retinoic acid and ROS, and this seems to underlie the functional roles of ALDHs in CSCs [101].

ALDH1 activity may be modulated by DKK-1, a Wnt antagonist, and ALDH1 can be involved in osteosarcoma cancer cell survival and resistance to chemotherapy. In this study, transcriptional activation of ALDH1 was dependent on the activation of the non-canonical Jun-mediated Wnt pathway, suggesting that signaling pathways other than those controlling self-renewal (e.g., Wnt/β-catenin signaling) can also participate in the modulation of ALDH activity [49]. Another study associated the resistance of Saos-2 and U2OS osteosarcoma cells to doxorubicin with activation of ALDH1/CD133-positive cells. This resistance phenotype was inhibited by forced expression of miR-143, which suggests that it may play a role in tumor suppression in osteosarcoma by counteracting stemness properties such as ALDH expression [61]. Retinal treatment preferentially affected ALDH-high cells by decreasing their proliferation, invasion capacity, and resistance to oxidative stress. These effects were more pronounced in highly metastatic osteosarcoma cells, accompanied by altered expression of metastasis-related genes and downregulation of Notch signaling markers [51].

Our previous experimental studies indicate that established osteosarcoma cell lines possess an Aldefluor-positive cell fraction that are *SOX2*-positive but *KLF4*-negative, and further enriched in CSCs isolated from spherical colonies [41]. Exposure of therapy-naïve cell lines to conventional drugs increased ALDH signaling as assessed by Aldefluor activity and *ALDH1A1*, *ALDH2* and *ALDH7A1* mRNA expression [50], while Wnt/β-catenin inhibition with the tankyrase inhibitor IWR-1 diminished the percentage of ALDH-positive cells and the previously mentioned ALDH isozymes [89]. Other authors have shown that miR-26a is downregulated in ALDH-positive ZOS and 143B cells, while its overexpression reduced ALDH activity via inhibition of another stem cell pathway, Jagged1/Notch [102].

Activity of ALDH can be inhibited with the FDA-approved drug disulfiram, which was used by the Weiss group to show its inhibitory effects in pulmonary metastasis formation in an immunocompetent Balb/c orthotopic mouse model, a result that was statistically equivalent to doxorubicin treatment [103]. This offers an alternative treatment route for osteosarcoma, in line with recent attempts of drug repurposing strategies based on gene expression signatures [104] and other in vitro modeling [105]. This was endeavored also for other drugs and tumor types [106], including the specific targeting of CSCs [107], and substantiates the possibility to refine the doses for the state-of-the-art approved drugs for osteosarcoma while decreasing their side-effects.

More recently, an elegant study using patient-derived xenograft models demonstrated that ALDH1-high xenografts with enhanced metastatic potential in vivo were sensitive to the β-catenin/transducin β-like protein 1 inhibitor tegavivint [52]. Moreover, it has been shown that ALDH activity was increased by the lncRNA THOR through enhanced SOX9 expression [108]. ALDH-positive cells were also sensitive to the apoptotic effects induced by the cell death ligand TRAIL and by leptomycin B [109]. Other studies also suggest that microRNAs may negatively regulate the expression of ALDH family members, such as miR-487b-3p and ALDH1A3, in vitro and in clinical samples [110], and miR-761 and ALDH1B1, in vitro and in vivo [111], which altogether substantiates the complex participation of ALDHs in the phenotypic behavior of osteosarcoma cells.

### 4.2. Chemoresistance Due to Enhanced Activity of ERK and AKT Survival-Related Signaling Pathways

The EGFR-Ras-Raf-MEK-ERK signaling network and the PI3K/PTEN/AKT signaling pathway controls cell growth and regulates cell survival, cell differentiation and apoptosis, thus being considered important targets for cancer therapy. Deregulation and oncogenic activation of these survival-related pathways is thus of utmost importance for CSCs and has been linked to stem cell features in other tumors [112]. Therefore, it is not surprising that the ERK and AKT pathways are upregulated in osteosarcoma and involved in tumorigenic features such as apoptosis resistance, in vivo tumorigenicity and EMT [113,114]. Some previous studies also pinpoint these two pathways as therapeutic targets [115,116]. Molecularly, the ERK pathway also seems to be involved in EMT and metastasis formation [117] and activated by the downregulation of p16 protein [118].

Several recent studies linked the activation of ERK and AKT pathways to stemness features in osteosarcoma. It is noteworthy that activation and modulation of these survival-related pathways strongly correlates with pluripotency-related transcriptional activity, depending also on the signaling cascades involved in cellular self-renewal, such as Wnt and Hedgehog, and correlates of EMT; they are also regulated by diverse types of microRNAs.

ERK is involved in the regulation of the Warburg effect via the physiological pathway regulator SLIT2/ROBO1 axis [119] and in cell proliferation and migration induced by the Wnt ligand WNT5A [120] and through Notch-induced ERK phosphorylation [121]. Other reports indicate that EMT in osteosarcoma is also regulated by ERK signaling [117,122]. ERK signaling also seems to participate in the acquisition of stemness features in osteosarcoma (expression of *CD24*, *CD90*, *CD133*, *Nanog*, *SOX2*, *Oct4*) mediated by miR-155; moreover, ERK inhibition with U0126 repressed expression of those markers [123]. Recently, Shimizu et al. reported that MEK inhibition with trametinib inhibited the cell cycle and induced apoptosis in non-adherent-growing U2OS cells. Moreover, trametinib decreased the size of primary tumors and circulating tumor cells in an in vivo mouse model [124].

The oncogenic long-non-coding MALAT1 was shown to activate the PI3K/Akt pathway via sponging miR-129-5p and promoted metastasis formation [125]. Activity of this pathway was also inhibited by the natural compound glaucocalyxin A, through the reduction in GLI1 activation and induction of apoptosis [126]. In the MNNG/HOS cell line, inhibition of the osteoblast regulator P2X7 hampered PI3K/Akt/GSK3β/β-catenin signaling and thereby inhibited stemness features and cell migration [127]. Moreover, PI3K/AKT also seems to be regulated by the lncRNA RNA FER1L4, via promoting apoptosis and suppressing EMT [128,129]. Recently, also TGF-β knockdown was associated to PI3K/Akt downregulation, suppressing viability and aggressiveness in osteosarcoma CSCs [130,131].

### 4.3. Chemoresistance Due to Altered Metabolism

Metabolic alterations occurring in cancer cells have been ascribed as an important hallmark of cancer [132]. In fact, despite that the specific characterization of metabolomics in CSCs has been scarce compared to regular cancer cells, diverse studies suggest that CSCs preferentially use glycolysis and have a declined oxidative phosphorylation activity. However, the location in which CSCs reside at the tumors (e.g., in normoxic vs. hypoxic regions) seems to be detrimental to the type of energy source that CSCs allocate to sustain their proliferative capacity and survival skills [133], which clearly suggests their intrinsic reprogramming capacity to adapt their bioenergetics response depending on the tumor microenvironment. Some studies specifically focused on the metabolic features of osteosarcoma CSCs are summarized below. For instance, Mizushima et al. suggest that indeed osteosarcoma CSCs follow the trend to be more aerobic glycolytic than to use oxidative phosphorylation, partly dependent on the antigen LIN28 [134].

Different types of mass spectrometry-based techniques have been used to explore the nature of osteosarcoma CSC metabolism. Zhong and colleagues used an ultra-high-performance liquid chromatography coupled with tandem Q-Exactive Orbitrap mass spectrometer to characterize the HOS-CSC isolated with the sphere-forming assay [135]. They found a significant upregulation of the metabolomics of several amino acids (alanine, aspartate, glutamate, arginine, proline, glutathione, cysteine and methionine) and a declined mitochondrial function and TCA cycle. Other authors used untargeted liquid chromatography with tandem mass spectrometry (LC–MS/MS) analysis to explore the metabolomics features of the osteosarcoma 143B and MG-63 spheres’ response to MTX [45]. They found that CSCs had alterations in the metabolomics of amino acid, fatty acid, energy and nucleic acid after treatment with MTX and emphasized the utility of mass spectrometry techniques to study the metabolomics features of CSCs. However, these authors did not specifically analyze whether the modulation of those metabolites upon MTX treatment had a pro- or anti-survival advantage to CSCs in that context.

3AB-OS-MG-63 has been used as an in vitro model to study osteosarcoma CSC metabolism. Palorini et al. analyzed the metabolic features of the 3AB-OS-selected CSCs and their parental MG-63 cells and extensively characterized the bioenergetics of CSCs. Compared to parental cells, it was demonstrated that 3AB-OS-CSC depend more on glycolytic metabolism, proliferate less when cultured in glucose-starvation conditions and have increased expression of lactate dehydrogenase A (LDHA). Moreover, their reduced mitochondrial respiration and fragmented mitochondria led the authors to suggest that CSCs possess metabolic similarities to normal stem cells [136]. Subsequent studies using this cell model substantiated the assumption that 3AB-OS-CSC rely more on glycolysis and MG-63 cells rely on glutamine oxidation. Moreover, cisplatin treatment in glutamine-depleted MG-63 resulted in additive inhibitory effects on cell survival, while promoting a pro-survival S-phase arrest in glucose-starved 3AB-OS-CSC. Of special note is the fact that when exposed to cisplatin in glucose-deprived conditions, CSCs switched their metabolic status, reprogramming to be more oxidative than glycolytic and to increase their mitochondrial functions [137]. These results demonstrate the plasticity of metabolic networks in CSCs and that contribute to circumvent cisplatin cytotoxicity.

Osteosarcoma, as a relatively undifferentiated bone sarcoma, retains some mesenchymal features, such as the capacity to respond to adipogenic stimuli. In this context, some authors explored the fatty acid metabolism of osteosarcoma CSCs using the anti-diabetic thiazolidinedione. This PPARγ agonist induced growth arrest and adipogenic differentiation in Sox2-high CSCs, via a mechanism involving cytoplasmic sequestration of the transcription factors SOX2 and YAP [138]. Other authors showed that the inhibition of TNIK (an essential factor for the transactivation of Wnt signal target genes) with NCB-0846 decreased the expression of stem cell genes (*SOX2*, *NANOG*, *OCT4*, *MYC*) and ALDH activity and also favored lipid biosynthesis, driving osteosarcoma cells’ differentiation into adipocyte-like cells, via induction of PPARγ [139]. These studies unveil the potential to modulate adipogenesis in osteosarcoma cells in order to affect their cell fate determination and promote their vulnerability to previously unexpected drugs that may be repurposed and used as new treatment strategies.

Accumulating evidence suggests that the anti-diabetic drug metformin may be specifically used to target osteosarcoma CSCs and to modulate their metabolic profile. Shang et al. found that metformin inhibited glucose uptake, lactate production and ATP production in HOS CSCs. Resistance of those cells to cisplatin was correlated to overexpression of the pyruvate kinase isoenzyme M2, with its downregulation reversing cisplatin resistance potentiated by metformin treatment [140]. Our own previous studies showed that metformin has deleterious effects in MNNG-HOS CSCs, potentiating low-dose DOX-induced cytotoxicity. Moreover, metformin induced mitochondrial stress by activating the energetic sensor AMPK and increasing [18F]-FDG uptake and lactate production in parental cells but not in quiescent CSCs [141]. Interestingly, Zhao et al. also observed activation of AMPK, which led to the reversal of the mTOR pathway and triggered autophagy. Metformin induced apoptosis in osteosarcoma CSCs through the collapse of the mitochondrial transmembrane potential, decreased ATP synthesis and increased ROS production [142]. Altogether, these studies suggest that osteosarcoma CSCs rely also on mitochondrial respiration for energy production and, when exposed to metformin, are captured in an energetic crisis. These metabolic alterations disturbed the homeostasis of stemness and pluripotency in the osteosarcoma CSCs both in vitro and in vivo, and corroborate an important role for metabolic modulators to chemosensitize this resistant cell subset within the tumors. Actually, more recently, a pre-clinical test using canine osteosarcoma CSCs also showed the capacity of metformin to inhibit mitochondrial function, by decreasing oxygen consumption and ATP production, while sensitizing canine CSCs to irradiation therapy [143]. Further studies are required for a better elucidation of the therapeutic potential of metabolic modulators and the mechanisms involved in the interference with stemness features leading to sensitivity to drugs in osteosarcoma CSCs.

### 4.4. Chemoresistance Due to Cell Cycle Arrest and Cellular Quiescence

Dormancy or quiescence in tumors may be derived from a defective angiogenic process, in which the expansion of tumor mass is limited due to the inability of recruitment of new and functional blood vessels, generating tumor masses poorly vascularized. This process associates with cell cycle arrest in which the blockade of tumor mass expansion results from the quiescent state of tumor cells. Microenvironmental stimuli or intracellular hits leading to increased cell proliferation may result in escape from dormancy and expansion of the tumor mass, leading to the emergence of clinically relevant disease [144]. Quiescence is molecularly regulated by cell cycle-related signaling [145], and by the tumor suppressors p53 and RB proteins, whose genetic alterations are detrimental in osteosarcomagenesis.

Quiescence is also a common characteristic of drug-resistant cells and has been linked with stem cell traits, since, like normal stem cells, CSCs are quiescent, slow-cycling cells and therefore circumvent the effects of high levels of intracellular reactive ROS, which accounts for their self-renewal capacity and drug resistance [146]. When entering a quiescent, cell cycle-arrested state, CSCs allocate themselves a period of time that may be used to activate and implement one or more of the survival signaling pathways mentioned in this review.

Quiescent cell populations found in osteosarcoma are dependent on angiogenic switches. Almog et al. identified a set of dormancy-associated microRNAs that regulated the phenotypic switch of dormant to fast-proliferating cells, specially miR-190 and miR-580. Moreover, a KHOS-24OS-based mouse model with angiogenic cells overexpressing miR190 remained quiescent during at least 120 days [147]. IGF2 or insulin exposure created an autophagic state of cellular dormancy in highly tumorigenic osteosarcoma cells cultured under serum-free conditions, which then conferred resistance to doxorubicin, cisplatin and methotrexate [148].

We observed that osteosarcoma CSC-enriched spheres were in a slow-proliferative state, being Ki-67-negative [41], along with a low uptake of the glucose analogue [18F]-FDG and accumulation of cells in the G0/G1 phase [40]. Avril et al. also identified *OCT4*/*SOX2*/*NANOG*-positive MNNG-HOS 3D spheres, which were arrested in the G0/G1 phase. Interestingly, the non-diving state of the spheres was not changed by MSC-conditioned media [149]. MiR-329 induced G0/G1 cell cycle arrest and inhibited cell proliferation and tumorigenicity in vivo, effects that seem to be mediated by the DNA repair protein Rad10 [150]. Other authors identified miR-34a, miR-93 and miR-200c as regulators of osteosarcoma dormancy, which could be delivered to fast proliferating Saos-2 and MG-63 cells with the nanocarrier dPG-NH2 to reduce their aggressiveness and migration abilities [151].

More recently, quantitative imaging techniques, such as holographic imaging cytometry, time-lapse microscopy and a contrast-based segmentation algorithm, were fine-tuned and used to monitor the transitions between angiogenic/non-angiogenic tumor states [152] and between diving/non-dividing cells, showing that non-dividing MG-63 cells did not constitute the CSC pool [153]. These techniques allow the observation of key cellular morphological behaviors that cancer cells reshuffle during their dormant and metastatic states, namely, altered cell motility speeds and cell cycle lengths, demonstrating that quiescent cells do not mandatorily represent stem cell subsets.

### 4.5. Chemoresistance Due to Enhanced DNA Repair

DNA-damaging agents, such as most conventional chemotherapeutics used in osteosarcoma treatment, elicit diverse types of lesions in the DNA molecules (e.g., single- and double-strand breaks). Cancer cells recognize those lesions and bypass the cytotoxic stress induced by anticancer agents, by activating various DNA repair pathways, such as nucleotide excision repair, base excision repair, homologous recombination repair and non-homologous end joining [154]. Several studies unraveled the molecular basis of these DNA repair pathways and provided a rationale for the development of DNA repair inhibitors, which have been demonstrating therapeutic benefits and synergizing with those DNA-damaging drugs [155,156]. The PARP inhibitor olaparib, for treating BRCA1 or BRCA2 mutated tumors, represents the first drug based on this principle [157]. Some studies suggest that a prompt activation of DNA damage response and enhanced DNA repair capacity are key contributors to CSCs’ increased resistance to therapy, due to their extraordinary ability to repair the genetic code compared with their offspring [158].

In osteosarcoma, suppression of Rad51, the key protein of DNA homologous recombination repair, correlated with cell cycle arrest and limited in vivo tumor growth, while also sensitizing osteosarcoma HOS cells to ionizing radiation and chemotherapy [159]. The nucleotide excision repair variants XPD rs13181 and rs1799793 are also related to higher event-free survival in osteosarcoma patients receiving neoadjuvant chemotherapy, and their expression provided a therapeutic advantage from cisplatin chemotherapy, probably by reducing DNA repair activity [160]. Other studies suggest that homologous recombination deficiency associates with MG-63 and ZK-58 cells sensitivity to the PARP inhibitor talazoparib alone or in combination with chemotherapeutics [161].

More recently, expression of the DNA damage repair inducer SIRT6 was associated with shorter overall survival in patients who received adjuvant chemotherapy. In vitro, SIRT6 overexpression contributed to doxorubicin resistance, but was blocked by the PARP inhibitor olaparib [162]. TH1579, an inhibitor of the MTH1 enzyme, which prevents the integration of oxidized nucleotides into DNA, decreased viability, cell cycle progression and induced apoptosis in osteosarcoma cells in vitro and in vivo [163]. Resistance to cisplatin was counteracted by downregulation of the DNA-dependent protein kinase catalytic subunit (DNA-PKcs) in PI3K/Akt/CD133+ cells, via a reduction in MARK2 [164], and also in combination with the checkpoint kinase 1 inhibitor AZD7762 [165] and the nucleotide excision repair pathway inhibitors NSC130813 and triptolide [166]. The role of small nucleolar RNAs has gained attention in recent years. Some of these RNAs (SNORD3A, SNORA13 and SNORA28) induce resistance to doxorubicin by modulating the expression of genes involved in DNA-damage sensing (GADD45A) and DNA repair (MYC) [167]. Other reports suggest that SNORA7A, modulated by the lncRNA H19, is oncogenic in osteosarcoma and correlates with poor patient survival [168].

Despite more studies being required to provide a more convincing understanding of the DNA repair pathways in osteosarcoma, promising pre-clinical results testing chemotherapy-potentiating DNA repair inhibitors in osteosarcoma and other tumors seems to hold potential for targeting both CSCs and their offspring. Moreover, considering that the DNA-damaging drugs doxorubicin and cisplatin are the most effective elected therapy for osteosarcoma [169], understanding their pharmacogenetic mechanisms may help to counteract their associated secondary side-effects and hold potential to development more tailored combinatorial therapies.

### 4.6. Enhanced Anti-Apoptotic Mechanisms

Programmed cell death or apoptosis occurs in diverse cellular phases, such as normal development, organogenesis, ageing or inflammatory response, and serves as a natural barrier to cancer. Apoptotic mechanisms involve a complex signaling cascade and are composed of both upstream regulators and downstream effector components [170]. Currently, two distinct, although tightly interconnected, signaling pathways control apoptotic cell death. In the intrinsic or mitochondrial pathway, the counterbalance between anti- (e.g., Bcl-2, Bcl-xL, Mcl-1) and pro-apoptotic (e.g., Bax, Bak) proteins of the Bcl-2 family determines the trigger to mitochondrial apoptosis [171,172]. Another distinct way of controlling apoptosis occurs due to the activation of cell-surface death receptors (e.g., DR3, DR5, TRAIL, TNF receptors), which are responsive to a diversity of death ligands (e.g., TRAIL, TNF, FAS) expressed by immunocompetent cells [172,173]. In case that the net chief signal is pro-apoptotic, the apoptotic program then culminates in the activation of normally latent proteases (effector caspases-3, -6 and -7), which will be responsible for the execution phase of apoptosis, in which the cell is progressively disassembled and its contents, so-called apoptotic bodies, engulfed by neighboring and phagocytic cells [174,175].

Osteosarcoma cells are no exception in what concerns evasion from apoptosis. Expression of key anti-apoptotic proteins, such as Bcl-2 [176], Bcl-xL [177] and survivin [178], was detected in cell lines and patient samples and correlated with enhanced cell survival and proliferation in vitro and also associated with poor prognosis and metastatic dissemination [179]. Conversely, activation of pro-apoptotic proteins, even if mediated by other survival-related molecules [180,181], promotes apoptosis in osteosarcoma cells. In fact, a plethora of studies reporting effects on proteins of the apoptotic signaling cascades is derived from the genetic or pharmacological modulation of several other signaling pathways, including self-renewal-related pathways. Nevertheless, experimental data suggest that direct inhibition of anti-apoptotic proteins may help to combat resistance to chemotherapy.

Despite that most chemotherapeutic drugs exert their biological effects through induction of apoptotic cell death, evasion from apoptosis seems exacerbated in CSCs [182,183]. In this regard, attempts have been made to inhibit the relevant anti-apoptotic proteins, namely, Bcl-2, Bcl-xL and Bcl-W, using the BH-3 mimetic ABT-737 [184,185,186]. These preliminary data provide a rationale for a wider exploratory use of inhibitors of anti-apoptotic proteins to target chemoresistance of tumor cells in osteosarcoma, as earlier suggested [187,188,189], as well as CSCs as previously endeavored in other tumor types [184,185,186,190].

### 4.7. Chemoresistance Conveyed by the Tumor Microenvironment—Hypoxia and Inflammation

The tumor microenvironment surrounding CSCs is detrimental for the preservation of their stemness state in the niche. For example, it has been shown that tumor-associated MSCs release extracellular vesicles containing pro-proliferative, anti-apoptotic and metastatic supportive molecules involving microRNAs and growth factors [191]. MSCs also revealed tropism towards osteosarcoma cells, favoring their aggressiveness, through the expression of chemotactic factors, such as MCP-1, GRO-α and TGF-β1, and transdifferentiation to cancer-associated fibroblasts (CAFs), which further increased the cytokine levels in the tumor microenvironment and promoted transendothelial cell migration [192]. Conversely, osteosarcoma cells themselves are also capable of inducing MSC differentiation into CAFs through Notch/Akt signaling [193]. Endothelial cells secreting exosomes are also promoters of osteosarcoma stemness through Notch signaling [194].

#### 4.7.1. Hypoxia

Poorly organized networks of vascularization among solid tumors may determine the existence of hypoxic zones. The low oxygen tension present in these areas, despite generating toxic ROS, provides selective pressure for tumor growth and survival advantage for aggressive cells [195]. The central mediator of hypoxia, HIF-1, activates the transcription of genes involved in key aspects of cancer biology, representing an important therapeutic target. Moreover, hypoxia is considered an independent prognostic indicator of poor outcome and risk for metastasis development in osteosarcoma [196,197].

Expression of key hypoxia-related markers has been observed in osteosarcoma and was related to drug resistance [198], down-regulation of Wnt/β-catenin [199] and maintenance of the CSC phenotype in three-dimensional conditions [200]. Osteosarcoma as a solid tumor is highly susceptible to hypoxia activated pro-drugs such as TH-302, which has been shown to cooperate with doxorubicin against osteosarcoma-induced bone destruction and limiting the formation of pulmonary metastases [201]. The leading role of hypoxia in metastatic osteosarcoma dissemination seems to be mediated by the HIF-1α-CXCR4 pathway axis, which plays a crucial role during osteosarcoma cell migration [202,203]. Overexpression of the long non-coding RNA (lncRNA) HIF-2α-promoter upstream transcript (HIF2PUT) has gained attention as a regulatory marker of hypoxia in osteosarcoma. HIF2PUT inhibited in vitro osteosarcoma CSC proliferation and migration, the sphere-forming ability of MG-63 cells and decreased the CD133-positive cells [62], while others observed that HIF2PUT overexpression inhibited CSC migration and invasion through HIF2α downregulation [204]. Moreover, its expression levels were positively correlated with *HIF-2α* expression levels in primary tumor samples, predicting poor prognosis [205].

More recently, it was demonstrated that microvesicles derived from MSCs contributed to U2OS proliferation and migration under hypoxic conditions, which was associated with AKT and HIF-1α expression [206]. Hypoxia was suggested to induce dedifferentiation of MNNG/HOS cells, expression of Oct-4, Nanog and CD133, accelerated sphere formation and tumorigenesis in vivo—effects that were counterbalanced by the mTOR inhibitor rapamycin, which also suppressed HIF-1α expression [207]. Further studies are required concerning the role of hypoxia and oxidative stress in osteosarcoma CSCs’ biological features, in particular those studies involving adequate in vivo models that may uncover unanticipated markers of therapeutic resistance, such as HIF-2α previously reported in a humanized orthotopic model [208].

#### 4.7.2. Inflammatory Networks

Multiple studies have indicated that MSCs produce soluble factors closely involved in cell proliferation. Consequently, these factors exert a proliferative and pro-inflammatory effect on cancer cells [209,210]. MSCs can also contribute to tumor progression by cooperating with the tumor cells to develop a suitable microenvironment [211].

Further, the MSCs can also transdifferentiate into CAFs that are considered essential stromal cells and crucial regulators for this favorable microenvironment creation and tumor progression [193,212]. By themselves, stromal cells are not malignant and maintain the tissue structure and function. However, when these cells acquire an active phenotype, they sustain cancer cell growth and tumor progression [213]. CAFs have a significant role in stimulating angiogenesis, cell proliferation, invasion and motility by releasing growth factors and cytokines, deregulating Notch and p53 signaling pathways, and producing matrix metalloproteinases [214]. Both MSCs and CAFs secrete several soluble factors, extracellular vesicles, chemokines and cytokines that stimulate tumor sustenance, growth and angiogenesis in osteosarcoma [215]. Within these factors, IL-6 plays a leading role in the inflammatory profile of this microenvironment.

Indeed, IL-6 is increasingly recognized as the soluble mediator linking chronic inflammation to cancer development, and its protein and mRNA are often overexpressed in serum and tumor samples from breast, bone, liver and colon cancers, both in humans and mice [216,217,218,219]. This cytokine secretion is also augmented by an increase in NF-kB expression and is described to have a paracrine effect on osteosarcoma cells via activation of the STAT3 pathway, which is a well-known activator of cells’ proliferation, enhancing tumor aggressiveness [215,220]. IL-6 is also described as responsible for the increased chemoresistance of human osteosarcoma cells [221] and exacerbates the invasive capacity of cells contributing to tumor development when secreted by CAFs. Furthermore, Cortini et al. found that IL-6 is responsible for CSC sphere growth, suggesting that it could be involved in the maintenance of the CSC population extremely involved in osteosarcoma relapse [215].

Other inflammatory mediators, such as TNF-α, IL-8, COX-2, are also encoded by NF-kB. In particular, when interacting with its receptors (TNFR1 and TNFR2), TNF-α induces cell survival and proliferation, an inflammatory response and an anti-apoptotic signal by NF-kB activation [214]. TNF-α is also an essential regulator of cancer-related inflammation by modulation of T cells, B cells and tumor-associated macrophages (TAMs) [222]. Specifically, several studies have reported TAMs as important immune cells implicated in various tumor-promoting tasks, including pro-inflammatory signaling, enhancement of angiogenesis, invasion, metastasis and therapy resistance [223,224]. Notably, the crosstalk between CAFs and T cells is reciprocal. Barnas et al. have described that activated T cells secrete factors that enhance the production of IL-6 by lung CAFs. Then, these activated fibroblasts induced the expression of IFN-γ and IL-17, both of which are known to impact the progression as well as the inhibition of tumor growth and metastasis [225].

TGF-β1 has also emerged as one of the most relevant cytokines secreted by osteosarcoma cells and a key player in self-renewal and maintenance of stemness features. It is described that the tumor maintains the stemness of MSC through the TGF/Smad3 pathway and that osteosarcoma cells, via TGF-β1 secretion, enhance the production of pro-tumorigenic cytokines, such as IL-6, in the nearby stroma [226]. Additionally, TGF-β was shown to increase ROS’ production in CAFs by the impairment of the respiratory transport chain, specifically acting on Complex IV via GS3K action. Furthermore, the Smad signaling contributes to maintaining ROS accumulation contributing to the inflammatory networks that sustain tumor progression [227].

Besides the normal functions of fibroblasts in matrix remodeling and secretion of ECM components, accumulating evidence identify CAFs as modulators of a tumor’s immune milieu. These activated fibroblasts operate towards a pro-inflammatory and immunosuppressive microenvironment because of reciprocal interactions between immune cells and CAFs mediated by the CAFs’ secretome. Despite many studies, the plasticity of CAFs and their contribution to chemoresistance and stemness features within tumors are still poorly understood. However, numerous lines of evidence imply CAFs as potential targets to new therapeutic approaches to inhibit tumor progression and therefore also warrant further investigation for osteosarcoma-directed targeting.

## 5. Immunotherapy in Osteosarcoma

Immunotherapy has been described as a promising strategy for advanced cancer treatment [228]. New therapies involving the application of tumor vaccines, immune modulators, genetically modified T cells, cytokines, immune checkpoint inhibitors or combination therapy can be used to decrease treatment side reactions, increase therapeutic effects and improve the quality of life of cancer patients [229,230].

Recently, multiple clinical trials have been conducted in various tumors to test the efficacy of immunomodulators, and several preclinical studies have also supported the application of immunotherapy in osteosarcoma, as previously reviewed [231]. Osteosarcoma is characterized by poor immune responses, which include low densities of tumor-infiltrating lymphocytes and increased anti-inflammatory responses within the tumor, leading to drug resistance and diminished overall patient survival [232]. Monoclonal antibodies, such as anti-cytotoxic T lymphocyte antigen-4 (CTLA-4), B7-H3, programmed cell receptor-1 (PD-1), and its ligand (PD-L1), have been developed to target immune checkpoints and have garnered widespread attention due to their excellent therapeutic effects on malignant tumors. However, most checkpoint inhibitors are less effective in treating solid tumors, including osteosarcoma [230,233]. PDL-1 and PD-1 expression have been considered potentially useful biomarkers for further studies [234]. However, anti-PD-1 antibodies, such as pembrolizumab, nivolumab and atezolizumab, showed limited therapeutic efficacy in osteosarcoma patients [234,235,236,237]. Lussier et al. revealed that T cells that infiltrated the osteosarcoma microenvironment could downregulate additional inhibitory receptors such as CTLA-4, which conspired to hinder tumor immunity [238]. They combined CTLA-4 with a PD-1 antibody in a K7M2 murine model of metastatic osteosarcoma, and the tumor progression was under control. Additionally, Helm et al. found that osteosarcoma mouse models treated with a combination of CTLA-4 and PD-1 immune checkpoint inhibitors showed a rise in CD8+ T cells, increasing the cytotoxicity in the tumor microenvironment and contributing to a better prognosis [239].

Recent research using chimeric antigen receptor (CAR) T cells has provided promising outcomes [240,241]. Their target is the immune checkpoint molecule B7-H3 (CD276), a tumor antigen that is significantly upregulated in osteosarcoma [242]. This molecule is described to play a crucial role in T cell function inhibition [243]. Despite the advantages of CAR T cell therapy and its increasing usage in clinical practice in lymphomas and acute lymphocytic leukemia [244], its applicability in solid tumors presents multiple challenges [245]. In terms of production, the viral transduction variation efficiency, as well as the high cost and time-consuming nature of the T cell-manufacturing procedure, is a problem. Moreover, another factor in the failure to combat solid tumors with CAR T cells is the restricted infiltration and poor persistence caused by a stiff osteoid bone tumor matrix and immunosuppressive components in the tumor microenvironment [231,246].

Increasing evidence suggests that CSCs play a critical role in innate and adaptive immunity, influencing the effectiveness of immunotherapy. The activation of stemness programs appears to limit antitumor immune responses by decreasing T cell infiltration and increasing the expression of immunosuppressive checkpoints, resulting in immunologically cold microenvironments [247,248]. Widespread negative associations between stemness and anticancer immunity have been described for most cancers, suggesting that targeting the stemness phenotype may render tumors more responsive to immune control [247,249]. Rainusso et al. identified a mechanism by which targeting HER2 with genetically modified T cells could eliminate osteosarcoma CSCs [250]. More recently, Shao et al. showed evidence that all-trans retinoic acid could indirectly limit CSC occurrence in osteosarcoma by the inhibition of the M2-like macrophages that activated the CSC phenotype [251]. Curcumol seems to inhibit the polarization of M2-like macrophages synergizing with cisplatin and reversing the drug-induced expression of ABCB1, ABCC1 and ABCG2 [252]. Guo et al. also demonstrated that a low CSCs score is correlated with increased immunocyte infiltration [249]. Overall, the tumor microenvironment is a problem in modern cancer research, which has a decisive function in the occurrence and progression of tumors. Its complete characterization, including extensive knowledge of the immune microenvironment, is crucial for immunotherapy implementation. which can contribute to patients who are refractory to therapy or who have relapsed, improving their prognosis.

## 6. Conclusions

**Figure 4:**
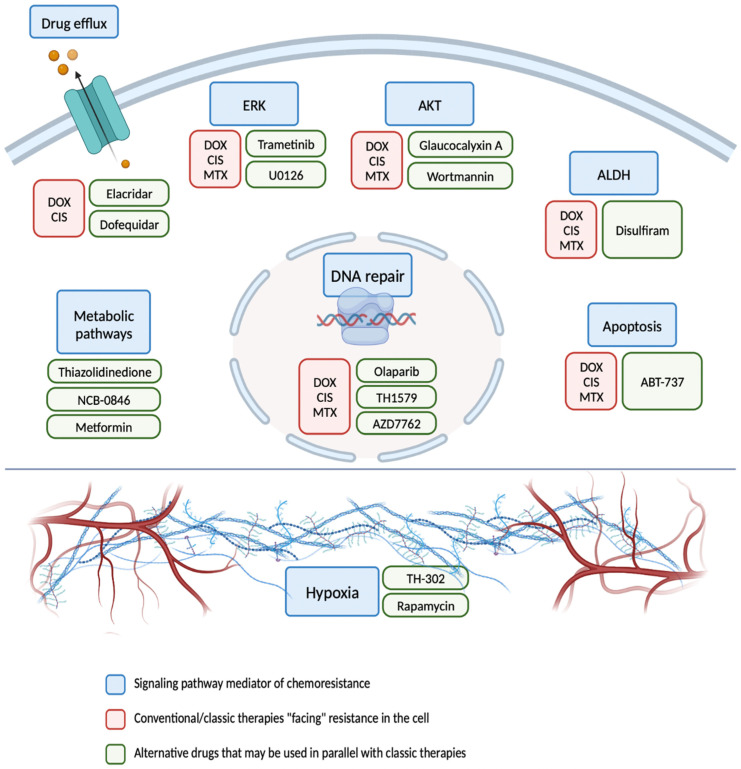
Graphical representation of the main pathways responsible for chemoresistance to the chemotherapies used in osteosarcoma (blue boxes). The conventional or classic drugs doxorubicin (DOX), cisplatin (CIS) and methotrexate (MTX) constitute the first-line treatment of osteosarcoma patients (red boxes) and often encounter tumor cell resistance mediated by those pathways. Alternative treatment strategies that can be applied in conjunction with classic drugs attempting to bypass drug resistance are represented in the green boxes.

The mechanisms of resistance explored in this revision confer resistance to doxorubicin, cisplatin and methotrexate, which are the main therapies used in osteosarcoma treatment. As mentioned before, this classic drug regimen still cannot be excluded from the main frontline treatments, since basically all the efforts made towards the discovery and development of alternative therapies have up to date failed in clinical trials. Moreover, as Harris and Hawkins have recently reviewed in *IJMS*, a number of drugs tested in diverse clinical trial phases have failed to significantly prolong the event-free survival or overall survival rates of poor responders to pre-operative chemotherapy or metastasis-presenting osteosarcoma patients (e.g., pirarubicin, pemetrexed, carboplatin, ifosfamide, etoposide, topotecan, thiotpeta, gemcitabine, L-alanosine, zoledronic acid, and several drugs targeting tyrosine kinases, among others) [253]. Nevertheless, a number of alternative drugs have been tested in osteosarcoma to target the resistance signaling pathways herein mentioned, as depicted in Figure 4, among many others. Fortunately, the rapid advances in the biological understanding of osteosarcoma, based on increasingly more robust pre-clinical models and access to rapid clinical testing, will certainly ameliorate the long-awaited outcomes of osteosarcoma patients.

The signaling pathways herein revisited clearly display inter-crosstalk and also modulate the activity and expression of other pathways involved in pluripotency and cell self-renewal. A precise detail of these latter pathways are a subject of discussion in another review in progress, but it is worth mentioning the relevance of Sox2, Nanog, Wnt, Notch and Stat3, along with diverse types of microRNAs. In general, the crosstalk with the pathways is harmonized to promote CSCs’ survival and aggressiveness, reinforcing their potential contribution to osteosarcoma resistance to chemotherapy and warranting further pre-clinical investigations focused on these special cell subsets.

## Figures and Tables

**Table 1:** Overview of the functional experimental techniques used to isolate CSCs in osteosarcoma, their technical principles, possible drawbacks and stem cell-related characteristics they have identified in the mentioned studies.

Method	Technical Principle	Expert Opinion	Stem Cell Features Found [References]
Sphere-forming assays	- most primitive and resilient cells survive the single-cell plating conditions in serum starvation culture systems; - suspended-growing conditions in non-adherent surfaces	- experimental variability introduced over the years hampers data comparison, because of the diversity of cell density plating, use of mitogens and media supplements; - sphere-formation does not necessarily correlate to enhanced tumorigenic ability; - sphere assay mainly enriches for a population of stem and progenitor cells, together with more differentiated cells	- expression of pluripotency-related markers [36,37,38,39,40]; - Wnt/β-catenin activation [41]; - resistance to chemotherapies [40,42,43,44,45,46]
Aldefluor™	flow cytometry analysis of the intracellular enzymatic activity of aldehyde dehydrogenases	- simple experimental kit, technically well-controlled; - needs specific flow cytometer filters which may not be available to all researchers; - some studies suggest that ALDH1A1 isoform is the major contributor of the positive phenotype, leaving the activity of other ALDH isoforms undetected; - can be difficult to separate a sufficient number of cells to conduct further biochemical experimental characterization	- sphere-forming MG-63 cells resistant to doxorubicin, cisplatin [47]; - stem cell marker expression and high tumorigenicity [48]; - DKK-1 expression [49]; - increased SOX2 expression [41]; - expression of ALDH isozymes, such as ALDH1A1 [50]; - metastatic dissemination [51,52]
Side-population	flow cytometry analysis of cellular extrusion of a vital dye (e.g., Hoechst-33342)	Critical parameters: - preparation of a single-cell suspension; - concentration and possible toxicity of the vital dye used; - incubation method namely temperature and duration; - type and concentration of the ABC transporters’ inhibitor used to establish the negative controls; - accuracy of the discrimination of debris, dead and single cells; - quality of flow cytometry filters; - can be difficult to separate a sufficient number of cells to conduct further biochemical experimental characterization	- tumorigenic capacity, expression of stemness-related markers [53]; - Wnt activation [54]; - CD44/Oct4 expression [55]; - sphere-formation, drug resistance, clonogenicity [56], correlation with ABCG2 expression [41]; - EIF4E/mTOR signaling and other genes involved in developmental processes [57]
Expression of specific surface markers (involved in e.g., cellular invasion, adhesion, and metastasis)	sorting of phenotypically dissimilar cancer cell subsets based on the expression of a membrane protein using flow cytometry;	- CSCs markers identified are based on those expressed by normal stem cells; - consider the possibility that sorted cancer cells reacquire their original markers; - inaccuracies in the sorting process itself; - CSC sorting in mesenchymal tumors based on surface marker expression has been more elusive than in other tumors and less consistent between research groups	- CD133 associated with poor prognosis and chemoresistance [36,58,59,60,61,62,63]; - CD29, CD90, CD105, CD44, ICAM-1, CD56 (mesenchymal signature) [64]; - CD117 [44,65,66]; - CBX3/ABCA5 [39]; - CD248 [67,68]; - CD271 [69,70,71]; - osteoblastic differentiation markers CD49b [72], CD24 [73]

## Data Availability

Not applicable.
